# Exploration of Molecular Structure, DFT Calculations, and Antioxidant Activity of a Hydrazone Derivative

**DOI:** 10.3390/antiox11112138

**Published:** 2022-10-28

**Authors:** Kundan Tayade, Gyu-Seong Yeom, Suban K. Sahoo, Horst Puschmann, Satish Balasaheb Nimse, Anil Kuwar

**Affiliations:** 1 School of Chemical Sciences, North Maharashtra University, Jalgaon 425001, India; 2 Department of Chemistry and Analytical Chemistry, Rajarshi Shahu Mahavidyalaya, Latur 413512, India; 3 Institute of Applied Chemistry and Department of Chemistry, Hallym University, Chuncheon 24252, Korea; 4 Department of Applied Chemistry, S.V. National Institute of Technology, Surat 395007, India; 5 Department of Chemistry, Durham University, Durham DH1 3LE, UK

**Keywords:** hydrazone Schiff base, antioxidant, X-ray diffraction, hydrogen bonding, DFT, ^1^H NMR, ^13^C NMR, ABTS assay, DPPH assay

## Abstract

The hydrazine derivatives are known to possess several biological activities including anticancer, antibacterial and anti-fungal, anticonvulsant, and antioxidant. This communication presents the synthesis, X-ray crystal structure analysis, DFT calculations, cell cytotoxicity, and antioxidant activity of the Schiff base 4,4′-((1E,1′E)-hydrazine-1,2-diylidenebis(ethan-1-yl-1-ylidene))bis(benzene-1,3-diol) (compound **2**). We have also isolated the side product compound **1** and characterized it using single X-ray crystallography. The crystal structure of compound **1** depicts that the ensuing C–H···N hydrogen bonding interaction is presented and discussed herein. In addition, the calculations using density functional theory (DFT) approximation supported by experimental ^1^H and ^13^C NMR studies on the key compound **2** are reported. The results of theoretical and experimental ^1^H and ^13^C NMR were concordant. The antioxidant activity of compound **2** was determined by using 2,2′-azinobis(3-ethylbenzthiazoline-6-sulfonic acid) (ABTS^•+^) radical cation assays and 2,2-diphenyl-1-picrylhydrazyl (DPPH) radical assay. Compound **2** demonstrated excellent antioxidant activity in ABTS assay (IC_50_ = 4.30 ± 0.21 µM) and DPPH assay (IC_50_ = 81.06 ± 0.72 µM) with almost no cytotoxicity below 25 µM.

## 1. Introduction

Hydrazone and its derivatives have played an integral and important role owing to the azomethine group, characterized by the triatomic structure C=N–N, enabling its use in various fields [1,2,3]. The hydrazine derivatives are known to possess a number of biological activities, including anticancer [4,5], antibacterial and anti-fungal [6,7], anticonvulsant [8], and antioxidant [9,10,11,12,13]. Not only hydrazones but also polyphenolic compounds are known for their excellent antioxidant activity [14,15,16]. Therefore, it was assumed that a hydrazine derivative with multiple hydroxyl groups should show prominent antioxidant activity.

Amongst various synthesis routes of hydrazones such as Japp-Klingemann reaction [17,18,19], coupling between aryl halides and non-substituted hydrazones [20,21], the coupling between hydrazines and ketones or aldehydes is one of the thoroughly used routes [22,23]. The nucleophilic nitrogen of the azomethine renders cation sensing and metal coordination ability to the structure containing azomethine group. Also, the imine carbon that has both electrophilic/nucleophilic characters allows availing the anion sensing ability. Due to the intrinsic nature of the C=N bond, the configurational isomerism can be attributed to making the molecular switches and machines by E→Z photoisomerization upon UV light irradiation. The E→Z isomerization can also be accompanied by forming a weak coordination complex followed by the simultaneous release of the cation. The E→Z isomerization results from two different mechanisms, rotation, and inversion [24,25,26].

**Scheme 1:**
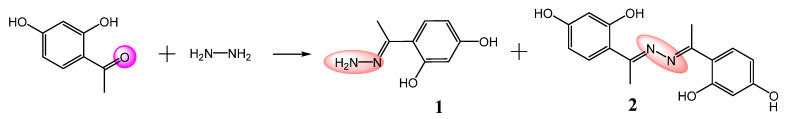
Synthesis of compounds **1** and **2**.

As a part of our ongoing research on the applications of organic motifs such as Schiff bases and their complexes in the field of chemosensors design and development [27,28,29,30], herein, we have discussed the synthesis and single X-ray crystallography studies of the Schiff bases, i.e., 4-[(1E)-1-hydrazinylideneethyl]benzene-1,3-diol (**1**) and 4,4′-((1E,1′E)-hydrazine-1,2-diylidenebis(ethan-1-yl-1-ylidene))bis(benzene-1,3-diol) (**2**) (Scheme 1). Also, the quantum chemical calculations of **2** have been performed using the density functional theory (DFT) and compared with the experimental results. Furthermore, we have performed DFT/B3LYP GIAO calculations of chemical shielding and report on an NMR characterization of compound **2**, using ^1^H and ^13^C NMR.

## 2. Materials and Methods

### 2.1. Materials and Instruments

The required chemicals were purchased from Sigma-Aldrich (Seoul, South Korea) and used as received. The A549 cells for the cytotoxicity study were purchased from the Korea cell line bank, Seoul, South Korea. 3-(4, 5-dimethyl thiazol-2yl)-2, 5-diphenyl tetrazolium bromide (MTB), fetal bovine serum (FBS), trypsin, and dulbecco Modified Eagle Medium (DMEM) were procured from Thermo Fisher Scientific, Waltham, MA, USA. The glass coverslips, cell culture plates, 96 well plates required for cell cytotoxicity assay were obtained from SPL Life Sciences, Seoul, Korea. The FT-IR spectra was recorded in the 4000–400 cm^−1^ region with a Shimadzu FTIR spectrophotometer (Shimadzu, Kyoto, Japan) using KBr pellet. The ^1^H NMR and ^13^C NMR spectra were recorded on a Bruker AVANCE-500 (400 MHz) spectrometer (Bruker, Billerica, UK) using DMSO-*d*_6_ as a solvent. The UV-Visible spectra were recorded on an Agilent UV-Visible spectrophotometer (Agilent Technologies, Santa Clara, CA, USA). Single-crystal X-ray diffraction experiments were performed using an Xcalibur, Sapphire3, Gemini ultra diffractometer equipped with an Oxford Cryosystems 700 Series (Agilent Technologies, Santa Clara, CA, USA) low-temperature apparatus operating at T = 120(2) K. Spectramax Plus 384 (Molecular Devices, San Jose, CA, USA) microplate reader was used for this study.

### 2.2. Synthesis of 4,4′-((1E,1′E)-hydrazine-1,2-diylidenebis(ethan-1-yl-1-ylidene))bis(benzene-1,3-diol)

The key compound **2** was synthesized by following slight modifications in the reported methods. At the same time, compound **1** was revealed as a byproduct formed in the reaction in trace quantities, and it was isolated [31,32]. The detailed procedure used for the synthesis of compound **2** is as follows. To a magnetically stirred solution of 2,4-dihydroxy acetophenone (0.304 g, 2 mmol) in ethanol, 5 mL solution of hydrazine hydrate (0.05 g, 1.0 mmol) dissolved in ethanol was added dropwise at an ambient temperature. After the complete addition of hydrazine hydrate, the reaction mixture was stirred and monitored by thin layer chromatography (hexane: acetone, 15%). A yellow solid appeared during the reaction. After completion of the reaction, the residue was filtered and washed with diethyl ether. The product was recrystallized from a mixture of hexane, ethyl acetate, and a few drops of *N*,*N*-dimethylformamide (DMF), affording desired product as pale yellow crystals. Yield: 67%, M.P. > 250 °C; FTIR (Nujol mull): 1605, 1569, 1460, 1377, 1299, 1252, 1159 cm^−1^; ^1^H NMR (400 MHz, DMSO-*d*_6_): δ (ppm) 2.45 (s, 6H, 2×-CH_3_), 6.31 (s, 2H, ArH), 6.37 (d, *J* = 7.2Hz, 2H), 7.59 (d, *J* = 8.7 Hz, 2H, ArH), 10.13 (brs, 2H, -OH), 13.58 (s, 2H, -OH,); ^13^C NMR (100 MHz, DMSO-*d*_6_): δ(ppm) 14.23, 102.90, 107.50, 111.22, 130.74, 161.57, 162.09, 166.63; ESI-MS (*m*/*z*), [C_16_H_16_N_2_O_4_+H^+^], Calculated: 301.32, Found: 301.46 (100%).

The diethyl ether used for the washing in the above reaction was allowed to evaporate slowly, which produced suitable crystals of compound **1**. Since this byproduct was obtained in trace quantity, we have not explored its detailed spectral characterization as it is a reported compound.

### 2.3. Single X-ray Crystallography

Single clear, colorless crystals of compounds **1** and **2** were obtained by slow evaporation from a water and ethanol medium mixture. This crystal with dimensions for compounds **1** and **2** were 0.26 × 0.17 × 0.12 mm^3^ and 0.85 × 0.24 × 0.14 mm^3^, respectively. Single-crystal X-ray diffraction data were measured using ω scans of 1.0° per frame for 9.0 s using MoK_α_ (λ = 0.71073 Å) radiation. The total number of runs and images was based on the strategy calculation from the program CrysAlisPro V1. 171.36.24. (Tokyo, Japan) [33]. The achieved resolution was θ = 26.997. Cell parameters were retrieved using the CrysAlisPro software and refined on 5507 reflections, 45% of the observed reflections. Data reduction was performed using the same software, which corrects Lorentz polarization. The final completeness is 99.90% out of 26.997 in θ. The absorption coefficient (µ) of compound **2** is 0.106, and the minimum and maximum transmissions are 0.72353 and 1.00000. Using Olex2, the structure was solved by Charge Flipping using the olex2.solve [34] structure solution program, and the model was refined with version 2013-2 of ShelXL [35] refinement package using Least Squares minimization [36]. All non-hydrogen atoms were refined anisotropically. The positions of hydrogens were calculated geometrically and refined using the riding model.

### 2.4. Theoretical Computations

All theoretical computations were made by using the computational code Gaussian 09W [37]. The initial geometry for the DFT calculations was based on the measured X-ray diffraction structure. We performed the geometry optimizations of compounds **1** and **2** using the DFT method with Becke’s three-parameter hybrid exchange–correlation functional (B3LYP) and the 6-31G (d,p) basis set in the gas phase. The harmonic vibrational frequencies and NMR chemical shifts were calculated at the same level of theory for the optimized structure. The chemical shifts were calculated using the Gauge-Invariant Atomic Orbital (GIAO) method using the 6-31G (d,p) basis set. The conductor-like polarizable continuum model (CPCM) was used to add the solvent effect (DMSO) to calculate the theoretical NMR spectra.

### 2.5. Determination of Antioxidant Activity

The antioxidant activity of compound **2** was measured using ABTS antioxidant assay and DPPH radical scavenging assay using ascorbic acid and quercetin as standard compounds. The antioxidant activity of compound **1** was not determined here, as it was isolated as a by-product in trace amounts. Furthermore, this compound is reported, and thus, it was believed unnecessary to pursue its antioxidant capacity.

#### 2.5.1. ABTS Antioxidant Assay

Equal amounts of 7 mM ABTS (2,2′-azino-bis(3-ethylbenzothiazoline-6-sulphonic acid)) stock solution and 2.45 mM potassium persulfate stock solution were mixed and stored at 0 °C for 12 h in the dark to produce the radical cation ABTS^•+^. The prepared radical cation ABTS^•+^ solution was diluted with methanol so that the UV absorption value was below 1.000 at 745 nm. The compound **2** and reference standards, including ascorbic acid and quercetin, were dissolved in methanol to obtain 2000 µM stock solutions. Then, these solutions were diluted with methanol to 1000, 500, 250, 125, 62.5, 31.3, 15.6, 7.81, 3.91, 1.95, 0.977, 0.488, 0.244, 0.122, and 0.0610 µM solutions. Then, 0.9 mL of the ABTS^•+^ solution and 0.1 mL of solutions of compound **2** or standard materials prepared above were mixed in a test tube and kept in the dark for 60 min. After 60 min incubation, the UV absorption at 745 nm was measured. All experiments were performed in triplicate. The radical scavenging rates were obtained from these UV absorption data, and the resulting IC_50_ values were calculated using Origin 8 (Northampton, MA, USA).

Percentage (%) of ABTS radical scavenging = [(A_0_ − A_1_)/A_0_] × 100 (1)

where A_0_ was the absorbance of the control, and A_1_ was the absorbance of the treated sample.

#### 2.5.2. Antioxidant Assay Using DPPH

The 100 µM DPPH stock solution was prepared in absolute methanol. The 2.0 mM stock solutions of compound **2** and the reference standards ascorbic acids and quercetin were also prepared in absolute methanol. Then, these solutions were diluted with methanol to 1000, 500, 250, 100, 50, 25, 12.5, 6.25, 3.13, 1.56, 0.781, 0.391, 0.195, 0.0977, 0.0488, 0.0244, and 0.0122 µM solutions. Afterwards, 0.7 mL of the DPPH solution and 0.7 mL of solutions of compound **2** or standard materials prepared above were mixed in a test tube and kept in the dark for 30 min. After 30 min incubation, the UV absorption at 517 nm was measured. All experiments were performed in triplicate. The radical scavenging rates were obtained from these UV absorption data, and the resulting IC_50_ values were calculated using Origin 8.

Percentage (%) of DPPH radical scavenging = [(A_0_ − A_1_)/A_0_] × 100 (2)

where A_0_ was the absorbance of the control, and A_1_ was the absorbance of the treated sample.

### 2.6. Cell Cytotoxicity Assay

The A549 cells (Colorectal carcinoma cell line) were used in the cytotoxicity assay (MTT (3-(4,5-dimethylthiazol-2-yl)-2,5-diphenyltetrazolium bromide) assay) of compound **2**. The A549 cells were grown in an incubator at 37 °C and 5% CO_2_ using DMEM media containing 2 mM glutamine and 10% FBS. Cells were trypsinized for seeding at 70–90% of cell confluency. About 7000 A549 cells per well were seeded in 96-well plates and incubated for 24 h at 37 °C in the presence of 5% CO_2_. Then, the media was replaced with the media containing compound **2** at various concentrations, including 0.1, 10, 25, and 50 µM, and incubated for another 24 h. Dimethyl sulfoxide (DMSO) was used as a vehicle. Hence, control wells were treated with equivalent volumes of DMSO. After 24 h incubation, the media was replaced with 200 µL of fresh media containing MTT solution and incubated for four hours at 37 °C. The absorbance was recorded at 570 nm to evaluate the cell viability. Each experiment was executed three times. Origin software was used for the data analysis.

## 3. Results and Discussion

### 3.1. Synthesis and Characterization of Compound ***2***

The simple condensation method was adopted to synthesize compound **2** (Scheme 1) [38,39,40,41]. The mixture isolated compound **1** was separated from **2** by simply washing the obtained product with diethyl ether. The reaction of 2,4-dihydroxy acetophenone with hydrazine hydrate in ethanol resulted in the formation of compound **2** with a 67% yield. The literature method [40] reported a yield of 68% for the un-crystallized final product. Hence, the synthesis method used for compound **2** here is comparable to the literature method considering the yield of 67% after crystallization. The ^1^H NMR (Figure S1), ^13^C NMR (Figure S2), mass (Figure S3), and IR (Figure S4) spectra confirmed the formation of compound **2**. The ^1^H NMR spectra show 2H of *ortho* -OH at 13.54 ppm, which was probably due to the hydrogen bonding with the imine -N atom, whereas the para -OH group appears at 10.15 ppm. The six methyl protons appear at 2.46 ppm. The ^13^C NMR spectra show a -CH_3_ carbon at 14.23 ppm and the -C=N carbon at 66.6 ppm. The mass spectra indicated a formation of the [M+H]^+^ peak at 301.46. The FT-IR spectrum of **2** showed characteristic peaks at 3450 cm^−1^ (-OH), 1606 cm^−1^ (-C=C- of C_6_H_5_ ring), 1560 cm^−1^ (-C=N-). Moreover, the UV-visible spectra of compound **2** demonstrated an absorption maxima (λ_max_) at 377 nm (Figure S5). Therefore, these results indicate the formation of compound **2**. As mentioned earlier, compound **1** is also formed during the reaction and was partially isolated. Compound **1** has been studied for various applications, including as a chemosensor [42,43]. Compound **1** was synthesized purposefully in the reported methods, unlike isolation from the filtrate as a side product presented in this article. Since the compound was isolated in trace amounts after the reaction workup, we did not monitor the reaction by NMR spectroscopy. Nonetheless, reaction monitoring by NMR spectroscopy would have allowed us to determine the ratio of the formation of compound **2** (major product) and compound **1** (minor product). Therefore, we have not conducted any spectroscopic measurements except for the single X-ray crystallography for compound **1**.

### 3.2. Single X-ray Crystallography and DFT Calculations

**Figure 1:**
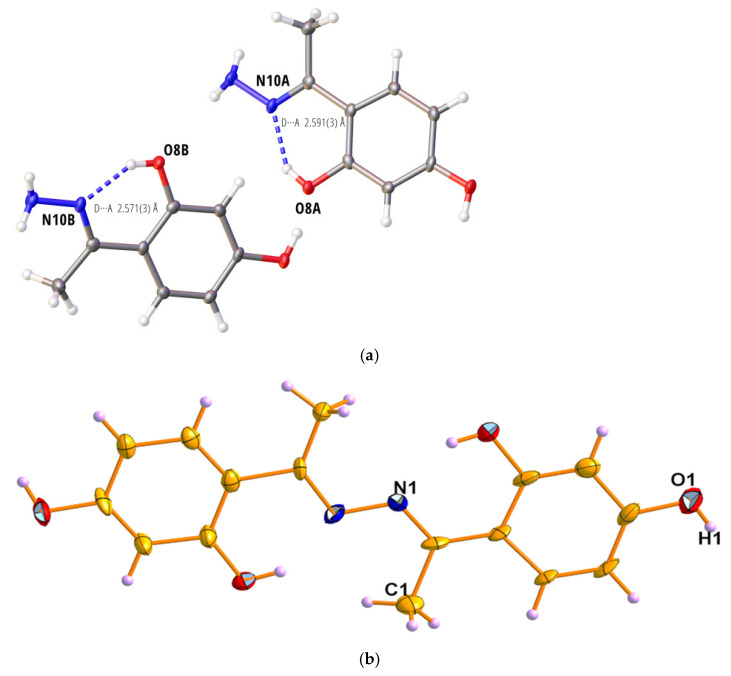
ORTEP diagrams of compounds (**a**) **1** and (**b**) **2**. The displacement ellipsoids are drawn at the 50% probability level.

The structures of compounds **1** and **2** were further characterized by X-ray crystallography. The CIF files for compounds **1** and **2** were deposited in the Cambridge Structure Database with CCDC No 1009469 and 1402230. The obtained ORTEP diagrams are shown in Figure 1a,b. Crystallographic data for compounds **1** and **2** are given in Table 1 (Tables S1 and S2, Figures S6–S9).

Hydrogen bonding is a major feature of the phenolic hydrazine types molecule. The phenolic hydrogen atom invariably forms an intramolecular hydrogen bond to the nitrogen atom of the hydrazine group, giving a six-membered ring. The D—A distance varies little between structures, with a maximum of 2.65 Å and a minimum of 2.51 Å [44]. In compound **1** (Figure 1a), the crystal chosen for the diffraction experiment turned out to be twinned with two clearly separated domains in the ratio 70/30. Only reflections of the stronger domain were integrated, resulting in good-quality data. The ‘unusual’ space group Ic is an alternative set of Cc. It was chosen because the beta angle in Cc would have been around 141°, which leads to an unwanted correlation in the refinement. The structure of **1** (C_8_H_10_N_2_O_2_) exhibits intra-molecular hydrogen bonding (Table 2) in each of the two independent molecules where the hydrogen atom of the phenolic hydroxyl group forms a strong O—H···N intra-molecular hydrogen bond with O···N distances of 2.571(3) Å and 2.591(3) Å. These distances are in the middle of the expected range for such hydrogen bonds. Each independent molecule forms a strong 2D hydrogen bonding network with symmetry-equivalent molecules of itself [45]. The D—A distances are 2.746(3) Å and 2.790(3) Å with a D—H···A angle of 158° [46].

**Figure 2:**
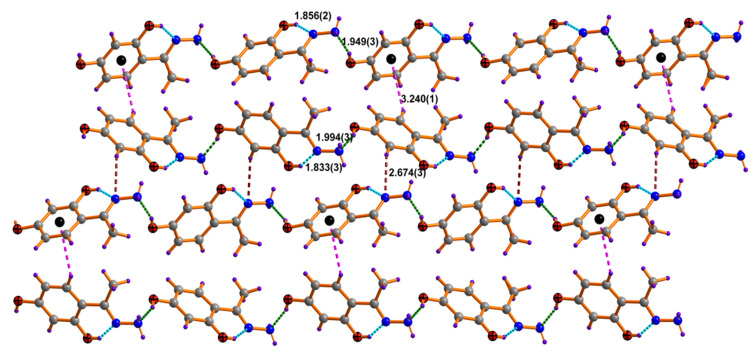
Packing diagram of compound **1** having two different molecules in the asymmetric unit.

Out of these two crystals, compound **1** shows an asymmetric unit intramolecular hydrogen bond distance for O–H···N=C interaction in the range of 1.833(3) to 1.856(2) Å. The corresponding O–H···N angle was found to be 145.2(1) and 145.7(1)° indicating comparatively strong intramolecular hydrogen bonding between these donor and acceptor atoms. Each molecule in the asymmetric unit independently interacts through C–H···N hydrogen bonding interaction to form a zig-zag pattern along the c-axis. The C–H···N distance was found to be 2.674(3). It is proposed that this C–H···N interaction may actually be present, or it might be simply a proximal artifact as a result of the other rigid bonding present in the crystal lattice. The hydrogen bonding interaction of nitrogen of H_2_N···O-H was found in the range of 1.949(3) to 1.994(3) Å. As depicted in the packing diagram of compound **1** (Figure 2), two molecules in an asymmetric unit are interconnected to each other via a weak CH···π(phenyl) interaction. The distance between the hydrogen atom and the centroid of the benzene ring was found to be 3.240(1) Å.

**Figure 3:**
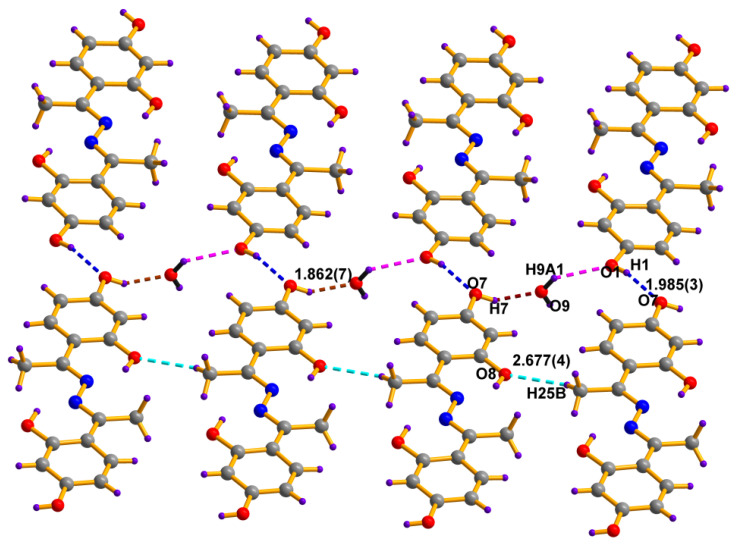
Packing diagram of compound **2** having two different molecules in the asymmetric unit.

Compound **2** crystallizes in the triclinic space group P-1, showing two almost identical molecules within the asymmetric unit. In both molecules, the dihedral angle passes through phenyl moiety, making an angle of 2.55(9)° and 0.75(13)°. The other geometrical parameters within the hydrazine molecule are observed as usual. Interestingly, the compound shows an intramolecular hydrogen bonding between the hydrazine nitrogen atom and the hydrogen atom of the hydroxyl group adjacent to it. These intramolecular distances are found in the range of 1.791(3) to (1.810(4) Å, and the corresponding angle O–H···N was 144.2(2)° to 147.1(2)°, which indicates a significantly strong intramolecular hydrogen bond between these donors and acceptors. The packing diagram shows two molecules in an asymmetric unit are interconnected to each other via a hydrogen bonding mediated by an H_2_O molecule (Figure 3). The water molecule interacts with the oxygen and hydrogen atoms of an adjacent molecule, forming a β-plated sheet along the c-axis. These sheets are arranged in an ababab fashion along the c-axis. The other weak interaction was observed between methyl hydrogen and hydroxyl oxygen atom H25···O8 2.677(4) Å with a bond angle of 148.6° (3). Both molecules in the asymmetric unit are arranged in a zig-zag manner, as shown in Figure 3.

**Figure 4:**
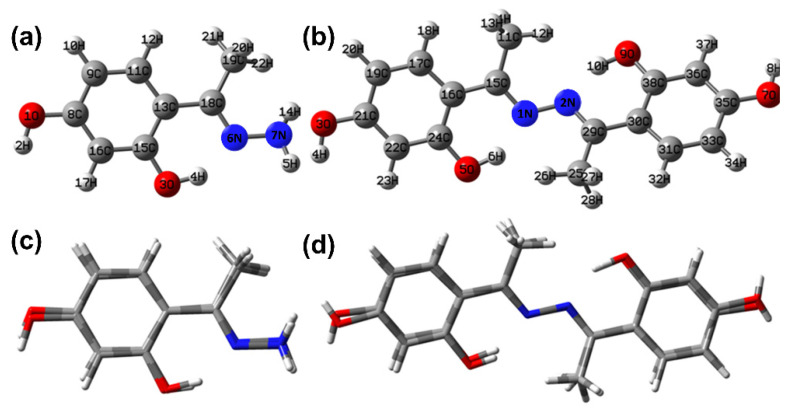
DFT computed the molecular structures of (**a**) compound **1** and (**b**) compound **2**. Atom-by-atom superimposition of (**c**) compound **1** and (**d**) compound **2** calculated over the X-ray structure.

The quantum mechanically computed structures of compounds **1** and **2** were obtained based on their crystallographic data at the 6.31G(d,p) level using the DFT/B3LYP method in the gas phase (Figure 4). The optimized geometry was checked as minima on the potential energy surfaces by calculating the vibrational frequency at the same level of theory, which showed no negative frequency. The global energy minimum obtained for compounds **1** and **2** were −570.8129 and −1029.7542 Hartree with the dipole moment of 2.6255 and 0.6732 Debye, respectively. The C1 symmetry point group was observed for both compounds. To make a direct comparison, the DFT computed geometric parameters of **1** and **2** are summarized in Table 3 with their corresponding experimental values obtained from the crystal structure. The data in Table 3 indicate the difference observed between the computed and experimentally determined bond lengths and angles of the compounds. The discrepancies can be explained by the fact that the DFT calculations are performed for a single molecule in the gas phase.

In contrast, the experimental values belong to the solid phase determined by X-ray diffraction. As shown in Figure 2 and Figure 3, the existence of intermolecular interactions in the solid phase, such as van der Waals interactions, hydrogen bonding, π···π and CH···π interactions, etc., resulted in the difference of bond parameters between the experimental and the calculated values [47]. Furthermore, to account for the accuracy of the theoretical approach, the DFT computed structures of **1** and **2** were superimposed with those obtained from X-ray crystallography, giving a root-mean-square error (RMSE) of 0.190 Å and 0.155 Å, respectively (Figure 4). These RMSE values indicate a good correlation between the calculated and experimental structures.

The observed ^1^H and ^13^C NMR (Figures S1 and S2) chemical shift values (in DMSO-*d*_6_) of compound **2** and the DFT computed values are summarized in Table 4. The plausible causes of errors might associate with the inaccuracy of DFT calculations, neglecting vibrational averaging [48].

### 3.3. Antioxidant Activity

Free radical scavenging is one of the most well-known methods for determining antioxidant activity [49,50]. The ABTS assay [51] and DPPH assay [52] are the commonly used radical scavenging assays to assess synthetic or natural compounds’ antioxidant potential. Here, we performed both ABTS and DPPH assays to evaluate the antioxidant potential of compound **2** in comparison to reference standards ascorbic acid and quercetin. The results of these assays are presented in Table 5 (Figures S10 and S11).

As shown in Table 5, the IC_50_ value of compound **2** (IC_50_ = 4.30 ± 0.21 µM), ascorbic acid (IC_50_ = 13.2 ± 0.45 µM), and quercetin (IC_50_ = 3.57 ± 0.54 µM), from ABTS assay indicate that compound **2** has a superior antioxidant activity to ascorbic acid. Compound **2** was also found to have comparable antioxidant activity to quercetin. Similar results were also observed in the DPPH assay. The radical-scavenging activity of compound **2** can be attributed to the in situ generation of resonance-stabilized phenoxide radical by the homolytic cleavage of the O-H bond during the ABTS and DPPH assays.

**Scheme 2:**
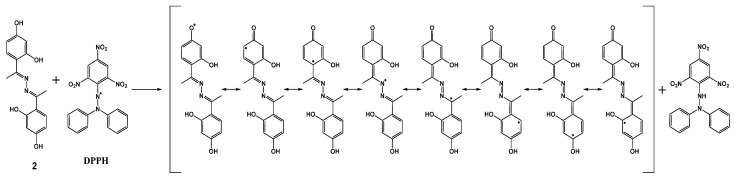
Proposed mechanism for the radical scavenging activity of compound **2**.

As depicted in Scheme 2, compound **2** can react with the ROS or RNS to scavenge the radical by using a homolytic cleavage of O-H bonds. The compound **2** radical that forms in this reaction is resonance stabilized. Hence, compound **2** shows excellent antioxidant activity.

### 3.4. Cell Cytotoxicity Assay

**Figure 5:**
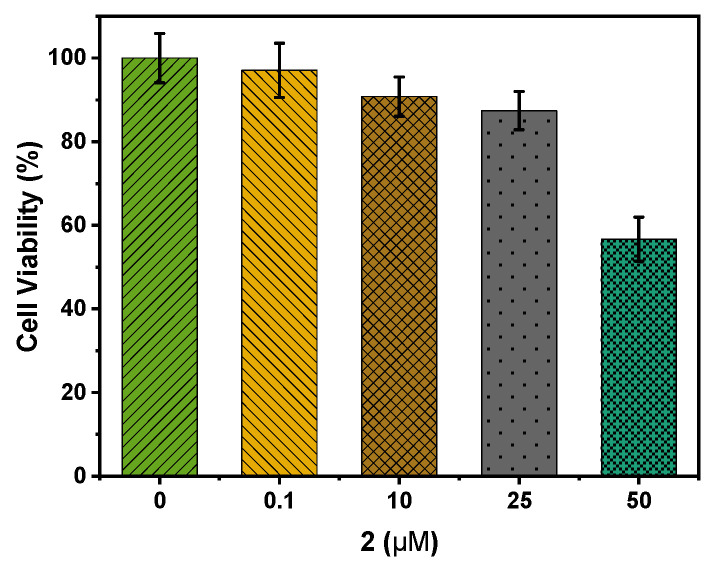
Cytotoxicity of compound **2** at 0, 0.1, 10, 25, and 50 µM was studied using A549 cells after 24 h in an MTT assay.

The MTT assay [53,54] allowed us to estimate the cytotoxicity of compound **2** after exposure of the A549 cells to the concentrations of 0.1, 10, 25, and 50 µM for 24 h with DMSO as a control. As shown in Figure 5, the results are shown as the percent cell growth compared to the control. Compound **2** did not show significant cell death even after 24 h of treatment at 0.1–25 µM concentrations. However, at 50 µM, compound **2** showed about a 40% decrease in cell growth compared to the control. Therefore, we believe that compound **2** has a high potential as a non-toxic antioxidant below the concentration of 25 µM.

## 4. Discussion

Here, we have presented the synthesis, characterization by single X-ray crystallography, antioxidant activity, and cell cytotoxicity of compound **2**. Compound **2** has been crystallized in a triclinic space group P-1 and contains two identical molecules within the asymmetric unit. The crystallography data also show the successful synthesis of compound **2**. As mentioned earlier, being a polyphenolic derivative, we expected compound **2** would show excellent antioxidant activity.

Reactive oxygen species (ROS) and reactive nitrogen species (RNS) are the most common free radicals containing either oxygen or nitrogen atoms in the molecules such as superoxide anion radical (O2^•^¯), hydroxyl radical (^•^OH), singlet oxygen (^1^O_2_), lipid radicals (LOO^•^), and nitric oxide (NO^•^) [55,56]. ROS and RNS are generated as by-products in several metabolic processes involving the oxidation of carbohydrates, fats, and proteins [57]. These free radical species are known to damage the cell membrane lipids, nucleic acids, proteins, and several other biomolecules that come in their vicinity [58]. Such radical reactions are usually controlled by enzymatic antioxidants such as catalase (CAT), glutathione peroxidase (GSHPx), superoxide dismutase (SOD), and peroxiredoxin I–IV (I–IV) [59]. However, the shortfall in the radical scavenging activity of these antioxidant enzymes leads to oxidative stress, resulting in various diseases, including cancer. Thus, the intervention by administering the antioxidant compounds is crucial to reduce oxidative stress [60]. The antioxidants can delay or inhibit cellular damage related to oxidative stress by inhibiting or quenching the free radicals [61].

Compound **2** presented in this article demonstrated comparable antioxidant activity with the standards such as ascorbic acid and quercetin in ABTS and DPPH antioxidants assays. The formation of resonance-stabilized compound **2** radical upon reaction with ROS endows it an excellent antioxidant activity. For any compound to be an excellent antioxidant, it must satisfy a minimum of two conditions: (i) it should show highly efficient radical scavenging activity, and (ii) it should be non-toxic. Hence, we have studied the cell cytotoxicity study of compound **2**. Compound **2** did not show any cell cytotoxicity at 25 µM. We believe that the synthesis of several derivatives of compound **2** and the evaluation of their radical scavenging activities can lead to an even more potent antioxidant hydrazine derivative.

## 5. Conclusions

In conclusion, we have presented the X-ray crystal structures and the DFT computed structures of the two Schiff base compounds **1** and **2**. Strong intramolecular hydrogen bonds along with the CH···π stabilized the molecules as observed from the crystal packing data. The DFT computations of **1** and **2** reproduced the structures obtained experimentally with some discrepancy due to the effects of non-covalent interactions in the solid-phase crystal-packing structure of **1** and **2**. The initial study on antioxidant activity using ABTS assay, DPPH assay, and cytotoxicity study revealed the high biological applicability of compound **2**. The polyphenolic nature of compound **2** along with the conjugated π-electron system endows it with a strong antioxidant activity. Further investigations in this direction by developing the derivatives of compound **2** are ongoing.

## Figures and Tables

**Table 1:** Crystallographic data for compounds **1** and **2**.

Parameter	Compound 1	Compound 2
CCDC	1009469	1402230
Formula	C_8_H_10_N_2_O_2_	C_16_H_18_N_2_O_5_
D*calc.*/g cm^−3^	1.441	1.408
µmm^−1^	0.106	0.106
Formula Weight	166.18	318.32
Color	clear colorless	Clear colorless
Shape	Irregular	Regular
Max Size/mm	0.85	0.26
Mid Size/mm	0.24	0.17
Min Size/mm	0.14	0.12
T/K	120(2)	293(2)
Crystal System	Monoclinic	Triclinic
Space Group	Ic	P-1
a/Å	16.431(3)	7.1880(17)
b/Å	4.7921(6)	10.347(2)
c/Å	21.042(4)	11.218(3)
α/°	90	70.033
β/°	112.40(2)	81.384(4)
γ/°	90	73.566
V/Å^3^	1531.8(5)	750.8(3)
*Z*	8	2
*Z′*	2.000	1.000
Θ*_min_*/°	2.682	1.935
Θ*_max_*/°	26.997	25.499
Measured Refl.	12,110	7515
Independent Refl.	3296	6653
Reflections Used	3116	4508
*R_int_*	0.0497	0.0266
Parameters	235	443
Restraints	2	3
Largest Peak	0.302	0.305
Deepest Hole	−0.291	−0.329
GooF	1.057	1.142
ω*R*2 (all data)	0.1200	0.1885
ω*R*2	0.1175	0.1756
*R*_1_ (all data)	0.0470	0.0951
*R*_1_	0.0446	0.0730

**Table 2:** Hydrogen bond geometry of compound **1**.

D	H	A	d(D-H)/Å	d(H-A)/Å	d(D-A)/Å	D-H-A/°
O8B	H8B	N10B	0.84	1.83	2.571(3)	145.7
O8A	H8A	N10A	0.84	1.86	2.591(3)	145.3
O1A	H1A	N11A^1^	0.84	1.95	2.746(4)	158.2
O1A	H1A	N11A^1^	0.84	1.95	2.746(4)	158.2
O1B	H1B	N11B^1^	0.84	2.00	2.790(4)	157.5
N11A	H11C	O8B	0.84	2.47	3.077(4)	129.0
^1^-1/2+X,3/2-Y,+Z						

**Table 3:** Selected structural parameters, including bond length (Å) and bond angle (°) by X-ray and theoretical calculations for compounds **1** and **2**.

Compound 1			Compound 2		
Bond Length (Å)	Expt.	DFT	Bond Length (Å)	Expt.	DFT
6N-4H	1.856	1.671	1N···6H	1.845	1.647
6N-3O	2.591	2.572	1N-5O	2.570	2.559
3O-4H	0.840	0.998	5O-6H	0.820	1.003
15C-3O	1.356	1.343	24C-5O	1.388	1.340
1O-2H	0.840	0.967	3O-4H	0.821	0.967
8C-1O	1.360	1.364	21C-3O	1.377	1.361
6N-7N	1.413	1.394	2N-1N	1.392	1.377
6N-18C	1.294	1.299	1N-15C	1.289	1.308
13C-18C	1.474	1.472	15C-16C	1.404	1.464
13C-11C	1.407	1.409	16C-17C	1.468	1.413
11C-9C	1.378	1.386	17C-19C	1.320	1.382
9C-8C	1.393	1.402	19C-21C	1.366	1.405
8C-16C	1.392	1.392	21C-22C	1.423	1.391
16C-15C	1.389	1.400	22C-24C	1.346	1.401
15C-13C	1.418	1.428	24C-16C	1.417	1.430
18C-19C	1.511	1.511	15C-11C	1.552	1.511
Bond angle (°)	Expt.	DFT	Bond angle (°)	Expt.	DFT
6N-4H-3O	145.28	147.9	1N-6H-5O	146.52	148.9
4H-3O-15C	109.46	106.4	6H-5O-24C	109.42	105.8
6N-18C-13C	117.51	117.7	1N-15C-16C	119.57	117.2
2H-1O-8C	109.51	109.0	4H-3O-21C	109.50	109.2
13C-18C-19C	120.78	121.4	16C-15C-11C	120.86	119.5
19C-18C-6N	121.70	120.9	11C-15C-1N	119.37	123.3

Expt., experimental; DFT, density function theory.

**Table 4:** Positions of atoms determined by X-ray crystallography and theoretical calculations for compound **2**.

Atom Position ^a^	Expt.	DFT	DFT (DMSO)	Atom Position ^a^	Expt.	DFT	DFT (DMSO)
10H	13.54	13.66	13.68	15C	166.63	161.96	164.21
32H	7.54	7.41	7.65	24C	162.09	158.99	158.55
34H	6.38	6.37	6.45	21C	161.57	153.95	154.65
37H	6.31	5.93	6.15	17C	130.74	126.00	127.39
8H	10.15	3.89	4.7	16C	111.22	109.81	110.25
-CH_3_	2.46	2.32	2.41	19C	107.5	101.73	102.2
				22C	102.9	97.87	98.3
				11C	14.23	13.88	14.67

^a^ Refer to Figure 4 for the atom positions; Expt., experimental; DFT, density function theory; DMSO, dimethyl sulfoxide.

**Table 5:** Antioxidant activity of compound **2** in comparison with ascorbic acid and quercetin.

Compound	ABTS Assay IC_50_, µM	DPPH Assay IC_50_, µM
Compound **2**	4.30 ± 0.21	81.06 ± 0.72
Ascorbic acid	13.2 ± 0.45	28.7 ± 0.65
Quercetin	3.57 ± 0.54	4.02 ± 0.058

## Data Availability

Data will be available from corresponding authors upon request.

## Supplementary Materials

The following supporting information can be downloaded at: https://www.mdpi.com/article/10.3390/antiox11112138/s1, Figure S1. ^1^H NMR spectrum of 2 in DMSO-*d*_6_; Figure S2. ^13^C NMR spectrum of 2 in DMSO-*d*_6_; Figure S3. Mass spectra of 2; Figure S4. IR spectra of 2; Figure S5. UV-visible spectra of 2; Table S1. Hydrogen bonds geometry of compound **1**; Figure S6. Data plots for diffraction data of compound **1**; Figure S7. Data plots for refinement data of compound **1**; Figure S8. Data plots for diffraction data of compound **2**; Figure S9. Data plots for refinement data of compound **2**; Table S2. Structure Quality Indicators; Figure S10. Results of ABTS assay for (a) Compound **2**, (b) ascorbic acid, (c) Quercetin; Figure S11. Results of DPPH assay for (a) Compound **2**, (b) ascorbic acid, (c) Quercetin.

## References

[1] Belskaya N.P., Dehaen W., Bakulev V.A. (2010). Synthesis and properties of hydrazones bearing amide, thioamide and amidine functions. ARKIVOC.

[2] Hammoud H., Elhabazi K., Quillet R., Bertin I., Utard V., Laboureyras E., Bourguignon J.J., Bihel F., Simonnet G., Simonin F. (2018). Aminoguanidine Hydrazone Derivatives as Nonpeptide NPFF1 Receptor Antagonists Reverse Opioid Induced Hyperalgesia. ACS Chem. Neurosci..

[3] Su X., Aprahamian I. (2014). Hydrazone-based switches, metallo-assemblies and sensors. Chem. Soc. Rev..

[4] Kaplánek R., Havlík M., Dolenský B., Rak J., Džubák P., Konečný P., Hajdúch M., Králová J., Král V. (2015). Synthesis and biological activity evaluation of hydrazone derivatives based on a Tröger’s base skeleton. Bioorg. Med. Chem..

[5] Palepu N.R., Premkumar J.R., Verma A.K., Bhattacharjee K., Joshi S.R., Forbes S., Mozharivskyj Y., Rao K.M. (2018). Antibacterial, in vitro antitumor activity and structural studies of rhodium and iridium complexes featuring the two positional isomers of pyridine carbaldehyde picolinic hydrazone ligand. Arab. J. Chem..

[6] He L.Y., Qiu X.Y., Cheng J.Y., Liu S.J., Wu S.M. (2018). Synthesis, characterization and crystal structures of vanadium (V) complexes derived from halido-substituted tridentate hydrazone compounds with antimicrobial activity. Polyhedron.

[7] Rawat P., Singh R.N., Niranjan P., Ranjan A., Holguín N.R.F. (2017). Evaluation of antituberculosis activity and DFT study on dipyrromethane-derived hydrazone derivatives. J. Mol. Struct..

[8] Dehestani L., Ahangar N., Hashemi S.M., Irannejad H., Masihi P.H., Shakiba A., Emami S. (2018). Design, synthesis, in vivo and in silico evaluation of phenacyl triazole hydrazones as new anticonvulsant agents. Bioorg. Chem..

[9] Anastassova N.O., Yancheva D.Y., Mavrova A.T., Kondeva-Burdina M.S., Tzankova V.I., Hristova-Avakumova N.G., Hadjimitova V.A. (2018). Design, synthesis, antioxidant properties and mechanism of action of new N, N′-disubstituted benzimidazole-2-thione hydrazone derivatives. J. Mol. Struct..

[10] Baldisserotto A., Demurtas M., Lampronti I., Moi D., Balboni G., Vertuani S., Manfredini S., Onnis V. (2018). Benzofuran hydrazones as potential scaffold in the development of multifunctional drugs: Synthesis and evaluation of antioxidant, photoprotective and antiproliferative activity. Eur. J. Med. Chem..

[11] Demurtas M., Baldisserotto A., Lampronti I., Moi D., Balboni G., Pacifico S., Vertuani S., Manfredini S., Onnis V. (2019). Indole derivatives as multifunctional drugs: Synthesis and evaluation of antioxidant, photoprotective and antiproliferative activity of indole hydrazones. Bioorg. Chem..

[12] Kareem H.S., Ariffin A., Nordin N., Heidelberg T., Abdul-Aziz A., Kong K.W., Yehye W.A. (2015). Correlation of antioxidant activities with theoretical studies for new hydrazone compounds bearing a 3, 4, 5-trimethoxy benzyl moiety. Eur. J. Med. Chem..

[13] Maltarollo V.G., de Resende M.F., Kronenberger T., Lino C.I., Sampaio M.C.P.D., da Rocha Pitta M.G., de Melo Rêgo M.J.B., Labanca R.A., de Oliveira R.B. (2019). In vitro and in silico studies of antioxidant activity of 2-thiazolylhydrazone derivatives. J. Mol. Graph. Model..

[14] Nimse S.B., Pal D. (2015). Free radicals, natural antioxidants, and their reaction mechanisms. RSC Adv..

[15] Cory H., Passarelli S., Szeto J., Tamez M., Mattei J. (2018). The role of polyphenols in human health and food systems: A mini-review. Front. Nutri..

[16] Xu D., Hu M.J., Wang Y.Q., Cui Y.L. (2019). Antioxidant activities of quercetin and its complexes for medicinal application. Molecules.

[17] Japp F.R., Klingemann F. (1887). Ueber Benzolazo-und Benzolhydrazofettsäuren. Ber. Dtsch. Chem. Ges..

[18] Japp F.R., Klingemann F. (1887). Zur Kenntniss der Benzolazo-und Benzolhydrazopropionsäuren. Ber. Dtsch. Chem. Ges..

[19] Japp F.R., Klingemann F. (1887). Ueber sogenannte gemischte Azoverbindungen. Ber. Dtsch. Chem. Ges..

[20] Wagaw S., Yang B.H., Buchwald S.L. (1999). A palladium-catalyzed method for the preparation of indoles via the Fischer indole synthesis. J. Am. Chem. Soc..

[21] Lefebvre V., Cailly T., Fabis F., Rault S. (2010). Two-step synthesis of substituted 3-aminoindazoles from 2-bromobenzonitriles. J. Org. Chem..

[22] Xia Y., Wang J. (2020). Transition-metal-catalyzed cross-coupling with ketones or aldehydes via N-tosylhydrazones. J. Am. Chem. Soc..

[23] Fulton J.R., Aggarwal V.K., de Vicente J. (2005). The use of tosylhydrazone salts as a safe alternative for handling diazo compounds and their applications in organic synthesis. Eur. J. Org. Chem..

[24] Horváth M., Cigáň M., Filo J., Jakusová K., Gáplovský M., Šándrik R., Gáplovský A. (2016). Isatin pentafluorophenylhydrazones: Interesting conformational change during anion sensing. RSC Adv..

[25] Filo J., Tisovský P., Csicsai K., Donovalová J., Gáplovský M., Gáplovský A., Cigáň M. (2019). Tautomeric photoswitches: Anion-assisted azo/azine-to-hydrazone photochromism. RSC Adv..

[26] Villada J.D., D’Vries R.F., Macías M., Zuluaga F., Chaur M.N. (2018). Structural characterization of a fluorescein hydrazone molecular switch with application towards logic gates. New J. Chem..

[27] Kuwar A.S., Fegade U.A., Tayade K.C., Patil U.D., Puschmann H., Gite V.V., Dalal D.S., Bendre R.S. (2013). Bis (2-hydroxy-3-isopropyl-6-methyl-benzaldehyde) ethylenediamine: A novel cation sensor. J. Fluoresce..

[28] Patil R., Moirangthem A., Butcher R., Singh N., Basu A., Tayade K., Fegade U., Hundiwale D., Kuwar A. (2014). Al^3+^ selective colorimetric and fluorescent red shifting chemosensor: Application in living cell imaging. Dalton Trans..

[29] Patil M., Park S.J., Yeom G.S., Bendre R., Kuwar A., Nimse S.B. (2022). Fluorescence’ turn-on’probe for nanomolar Zn (ii) detection in living cells and environmental samples. New J. Chem..

[30] Lee J.S., Warkad S.D., Shinde P.B., Kuwar A., Nimse S.B. (2020). A highly selective fluorescent probe for nanomolar detection of ferric ions in the living cells and aqueous media. Arab. J. Chem..

[31] Larsen D., Kietrys A.M., Clark S.A., Park H.S., Ekebergh A., Kool E.T. (2018). Exceptionally rapid oxime and hydrazone formation promoted by catalytic amine buffers with low toxicity. Chem. Sci..

[32] Newkome G.R., Fishel D.L. (1966). Synthesis of simple hydrazones of carbonyl compounds by an exchange reaction. J. Org. Chem..

[33] Agilent Technologies UK Ltd Crysalispro Software System, V1. 171.36.24. https://www.agilent.com/chem/.

[34] Dolomanov O.V., Bourhis L.J., Gildea R.J., Howard J.A., Puschmann H. (2009). OLEX2: A complete structure solution, refinement and analysis program. J. Appl. Crystallogr..

[35] Bourhis L.J., Dolomanov O.V., Gildea R.J., Howard J.A., Puschmann H. (2015). The anatomy of a comprehensive constrained, restrained refinement program for the modern computing environment–Olex2 dissected. Acta Crystallogr. A.

[36] Sheldrick G.M. (2008). A short history of SHELX. Acta Crystallogr. A.

[37] Frisch M.J., Trucks G.W., Schlegel H.B., Scuseria G.E., Robb M.A., Cheeseman J.R., Scalmani G., Barone V., Mennucci B., Petersson G.A. (2009). Gaussian 09, Revision E.01.

[38] Jang H.J., Kang J.H., Yun D., Kim C. (2018). A multifunctional selective “turn-on” fluorescent chemosensor for detection of Group IIIA ions Al^3+^, Ga^3+^ and In^3+^. Photochem. Photobiol. Sci..

[39] Torawane P., Tayade K., Bothra S., Sahoo S.K., Singh N., Borse A., Kuwar A. (2016). A highly selective and sensitive fluorescent ‘turn-on’chemosensor for Al^3+^ based on CN isomerisation mechanism with nanomolar detection. Sens. Actuators B Chem..

[40] Irmi N.M., Purwono B., Anwar C. (2021). Synthesis of Symmetrical Acetophenone Azine Derivatives as Colorimetric and Fluorescent Cyanide Chemosensors. Indones. J. Chem..

[41] Marcos M., Melendez E., Serrano J.L. (1983). Synthesis and Mesomorphic Properties of Three Homologous Series of 4, 4′-Dialkoxy-α, α′-Dimethylbenzalazines A Comparative Study (I). Mol. Cryst. Liq. Cryst..

[42] Said A.I., Georgiev N.I., Bojinov V.B. (2019). The simplest molecular chemosensor for detecting higher pHs, Cu^2+^ and S^2-^ in aqueous environment and executing various logic gates. J. Photochem. Photobiol. A Chem..

[43] Said A.I., Georgiev N.I., Bojinov V.B. (2022). Low Molecular Weight Probe for Selective Sensing of PH and Cu2+ Working as Three INHIBIT Based Digital Comparator. J. Fluoresc..

[44] Butcher R.J., Bendre R.S., Kuwar A.S. (2005). 2-Formylthymol oxime. Acta Cryst. Sect. E.

[45] Butcher R.J., Bendre R.S., Kuwar A.S. (2007). 6,6-Diisopropyl-3,3′-dimethyl-2,2′-azinodiphenol. Acta Cryst. Sec. E.

[46] Baharudin M.S., Taha M., Ismail N.H., Shah S.A.A., Yousuf S. (2012). N′-[(E)-2-Hydroxy-5-methoxybenzylidene]-2-methoxybenzohydrazide. Acta Cryst. Sec. E.

[47] Sahoo S.K., Sharma D., Bera R.K. (2012). Studies on molecular structure and tautomerism of a vitamin B6 analog with density functional theory. J. Mol. Mod..

[48] Dračínský M., Kaminský J., Bouř P. (2009). Relative importance of first and second derivatives of nuclear magnetic resonance chemical shifts and spin-spin coupling constants for vibrational averaging. J. Chem. Phys..

[49] Lee J.S., Song I.H., Shinde P.B., Nimse S.B. (2020). Macrocycles and Supramolecules as Antioxidants: Excellent Scaffolds for Development of Potential Therapeutic Agents. Antioxidants.

[50] Lee J.S., Song I.H., Warkad S.D., Yeom G.S., Shinde P.B., Song K.S., Nimse S.B. (2022). Synthesis and evaluation of 2-aryl-1 H-benzo [d] imidazole derivatives as potential microtubule targeting agents. Drug Dev. Res..

[51] Thongsuk P., Sameenoi Y. (2022). Colorimetric determination of radical scavenging activity of antioxidants using Fe3O4 magnetic nanoparticles. Arab. J. Chem..

[52] Blois M.S. (1958). Antioxidant determinations by the use of a stable free radical. Nature.

[53] Song I.H., Torawane P., Lee J.S., Warkad S.D., Borase A., Sahoo S.K., Nimse S.B., Kuwar A. (2021). The detection of Al^3+^ and Cu^2+^ ions using isonicotinohydrazide-based chemosensors and their application to live-cell imaging. Mat. Adv..

[54] Yeom G.S., Song I.H., Park S.J., Kuwar A., Nimse S.B. (2022). Development and application of a fluorescence turn-on probe for the nanomolar cysteine detection in serum and milk samples. J. Photochem. Photobiol. A Chem..

[55] Martínez M.C., Andriantsitohaina R. (2009). Reactive nitrogen species: Molecular mechanisms and potential significance in health and disease. Antiox. Red. Signal..

[56] Nosaka Y., Nosaka A.Y. (2017). Generation and detection of reactive oxygen species in photocatalysis. Chem. Rev..

[57] Schieber M., Chandel N.S. (2014). ROS function in redox signaling and oxidative stress. Cur. Biol..

[58] Wang X., Wang W., Li L., Perry G., Lee H.G., Zhu X. (2014). Oxidative stress and mitochondrial dysfunction in Alzheimer’s disease. Biophys. Acta Mol. Basis Dis..

[59] Sosa V., Moliné T., Somoza R., Paciucci R., Kondoh H., LLeonart M.E. (2013). Oxidative stress and cancer: An overview. Ageing Res. Rev..

[60] Young I.S., Woodside J.V. (2001). Antioxidants in health and disease. J. Clin. Pathol..

[61] Tan B.L., Norhaizan M.E., Liew W.P.P., Sulaiman Rahman H. (2018). Antioxidant and oxidative stress: A mutual interplay in age-related diseases. Front. Pharmacol..

