# Supplementary material for: Exploration of Molecular Structure, DFT Calculations, and Antioxidant Activity of a Hydrazone Derivative
Source: Antioxidants (Basel). 2022 Oct 28;11(11):2138. doi: 10.3390/antiox11112138 (PMC9686989; doi:10.3390/antiox11112138)
Supplement: Supplementary file 1 [file antioxidants-11-02138-s001.zip › antioxidants-1973435-supplementary.pdf]

Supporting Information

Exploration of molecular structure, DFT calculations, and antioxidant activity of a hydrazone derivative

Kundan Tayade [2,5,†], Gyu Seong Yeom [1,†], Suban K Sahoo [3], Horst Puschmann [4], Satish Balasaheb Nimse [1,*] and Anil Kuwar [2,*]

[1] Institute of Applied Chemistry and Department of Chemistry, Hallym University, Chuncheon 24252, Korea
[3] Department of Applied Chemistry, S.V. National Institute of Technology, Surat-395007, (Gujarat), India
[4] Department of Chemistry, Durham University, Durham, DH1 3LE, UK
[5] Department of Chemistry and Analytical Chemistry, Rajarshi Shahu Mahavidyalaya, Latur 413512, (MS), India

* Correspondence: satish_nimse@hallym.ac.kr (S.B.N.); kuwaras@gmail.com (A.K.)
† These authors contributed equally to this work. Hence, both should be considered as first authors.

Sr. No.	Content	Page no.
1	**Figure S1**. ^{1}H NMR spectrum of **2** in DMSO-d_6.	S2
2	**Figure S2**. ^{13}C NMR spectrum of **2** in DMSO-d_6.	S3
3	**Figure S3**. Mass spectra of **2**	S3
4	**Figure S4**. IR spectra of **2**	S4
5	**Figure S5**. UV-visible spectra of **2**	S4
6	**Table S1.** Hydrogen bonds geometry of compound **1**.	S5
7	**Figure S6.** Data plots for diffraction data of compound **1**.	S5
8	**Figure S7.** Data plots for refinement data of compound **1**.	S6
9	**Figure S8.** Data plots for diffraction data of compound **2**.	S6
10	**Figure S9.** Data plots for refinement data of compound **2**.	S7
11	**Table S2.** Structure Quality Indicators.	S7
12	**Figure S10.** Results of ABTS assay for **a)** Compound **2**, **b)** ascorbic acid**, c)** Quercetin.	S8
13	**Figure S11.** Results of DPPH assay for **a)** Compound **2**, **b)** ascorbic acid**, c)** Quercetin.	S8

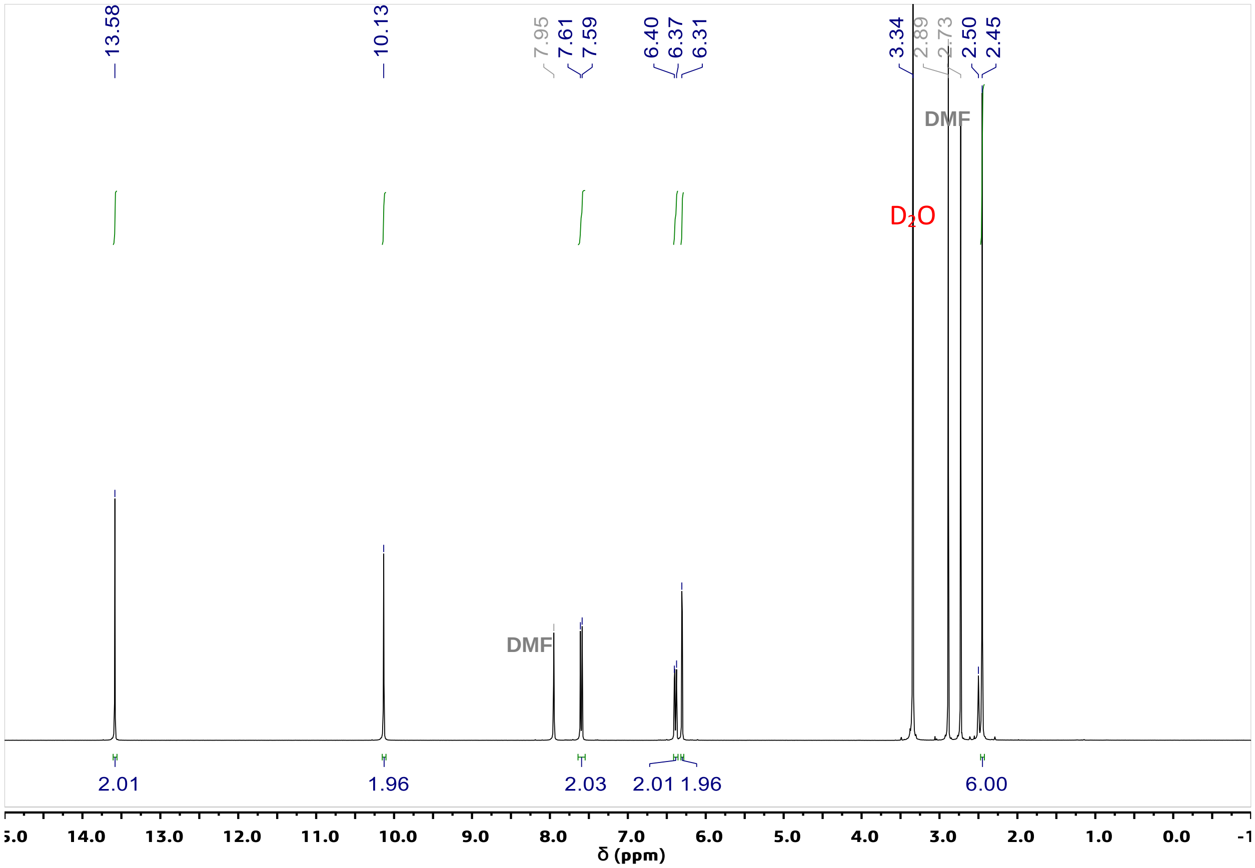

Figure S1. ^{1}H NMR spectra of **2** in DMSO-d_6.

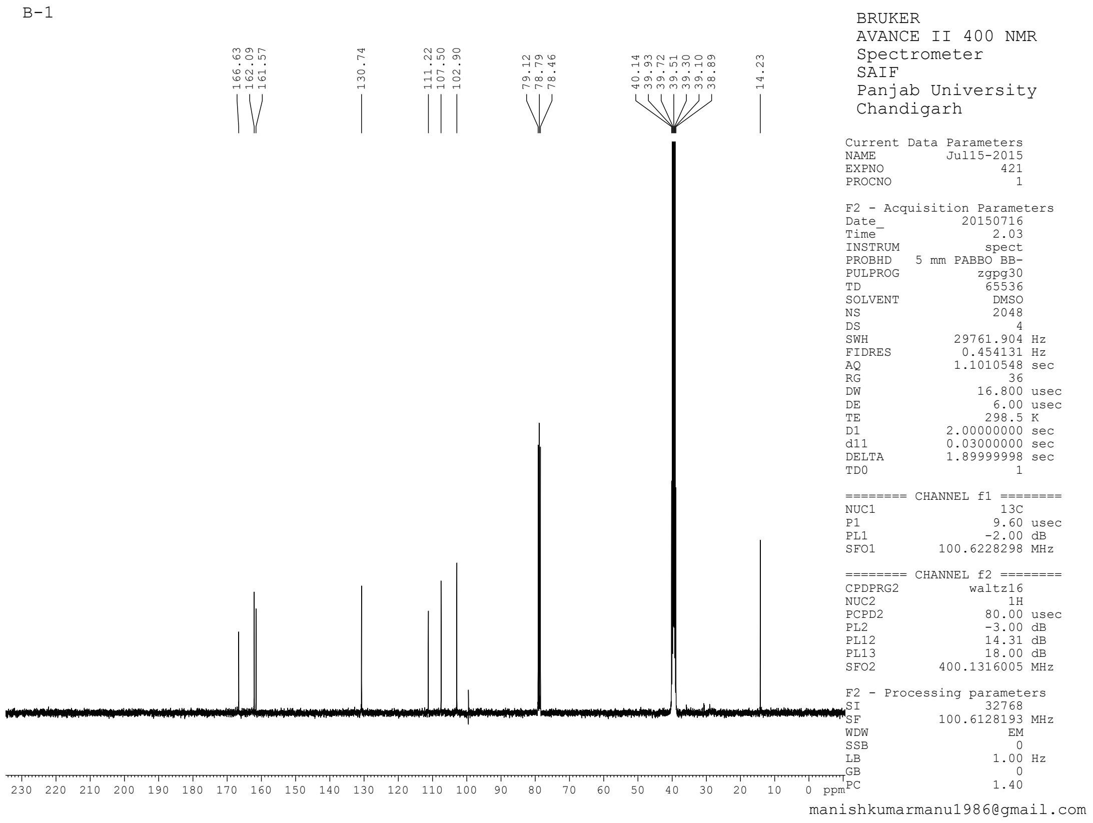

Figure S2.^{13}C NMR spectra of **2** in DMSO-d$_6$.

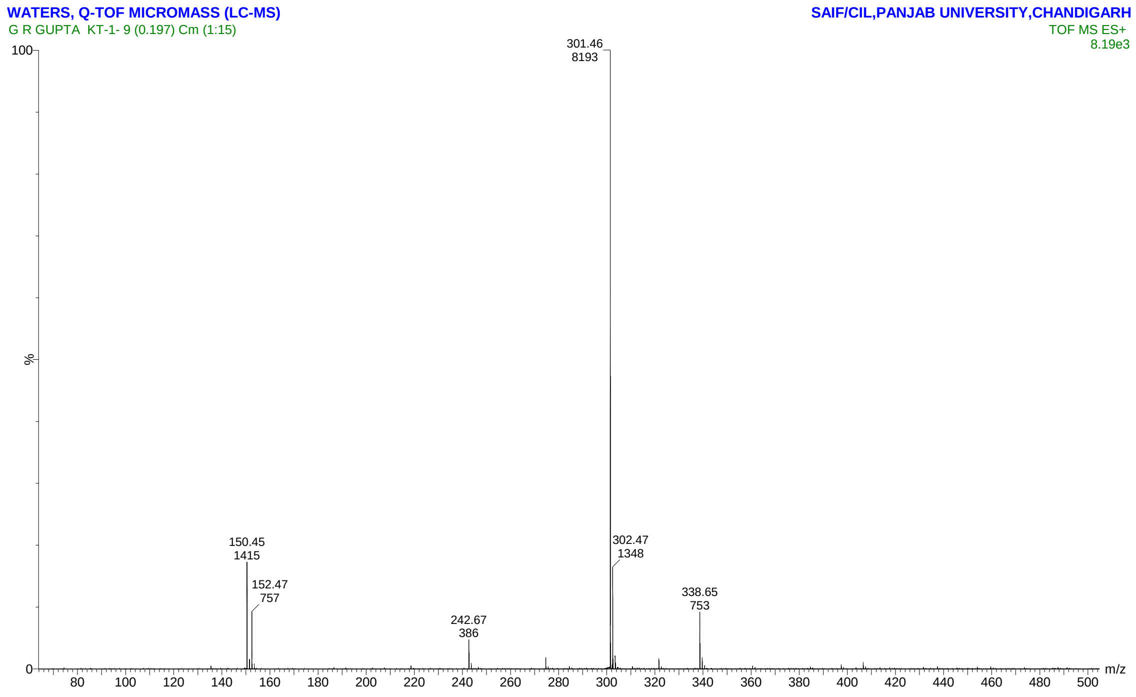

Figure S3. Mass spectra of compound **2**.

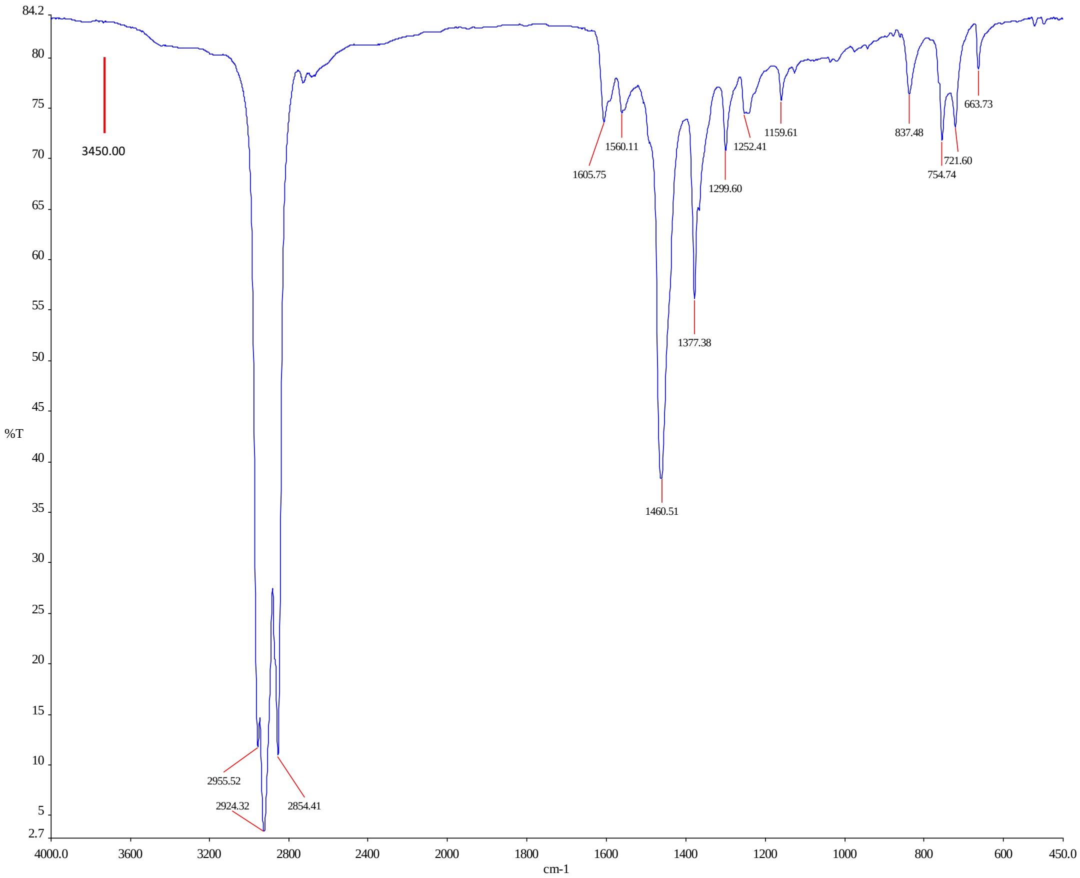

Figure S4: FT-IR spectra of compound **2**.

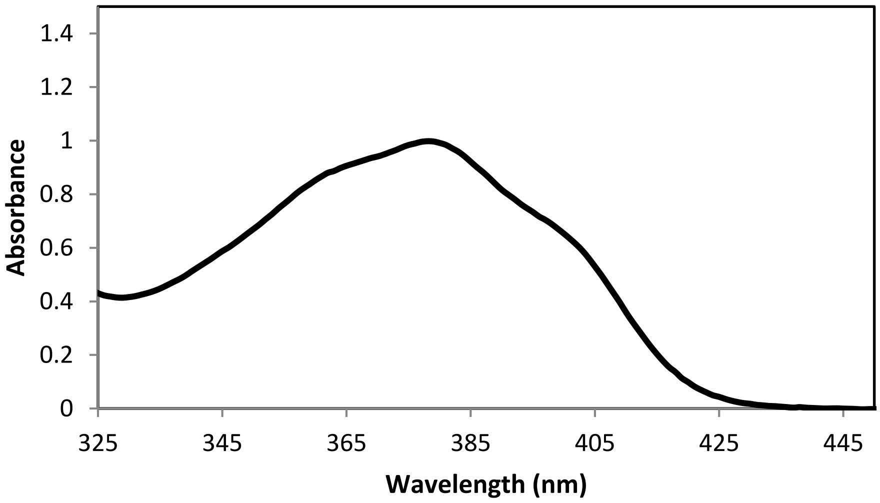

Figure S5: UV-visible spectra of compound **2**.

Table S1. Hydrogen bonds geometry of compound **1**.

D	H	A	d(D-H)/Å	d(H-A)/Å	d(D-A)/Å	D-H-A/°
O8B	H8B	N10B	0.84	1.83	2.571(3)	145.7
O8A	H8A	N10A	0.84	1.86	2.591(3)	145.3
O1A	H1A	N11A[1]	0.84	1.95	2.746(4)	158.2
O1A	H1A	N11A[1]	0.84	1.95	2.746(4)	158.2
O1B	H1B	N11B[1]	0.84	2.00	2.790(4)	157.5
N11A	H11C	O8B	0.84	2.47	3.077(4)	129.0

[1]-1/2+X,3/2-Y,+Z

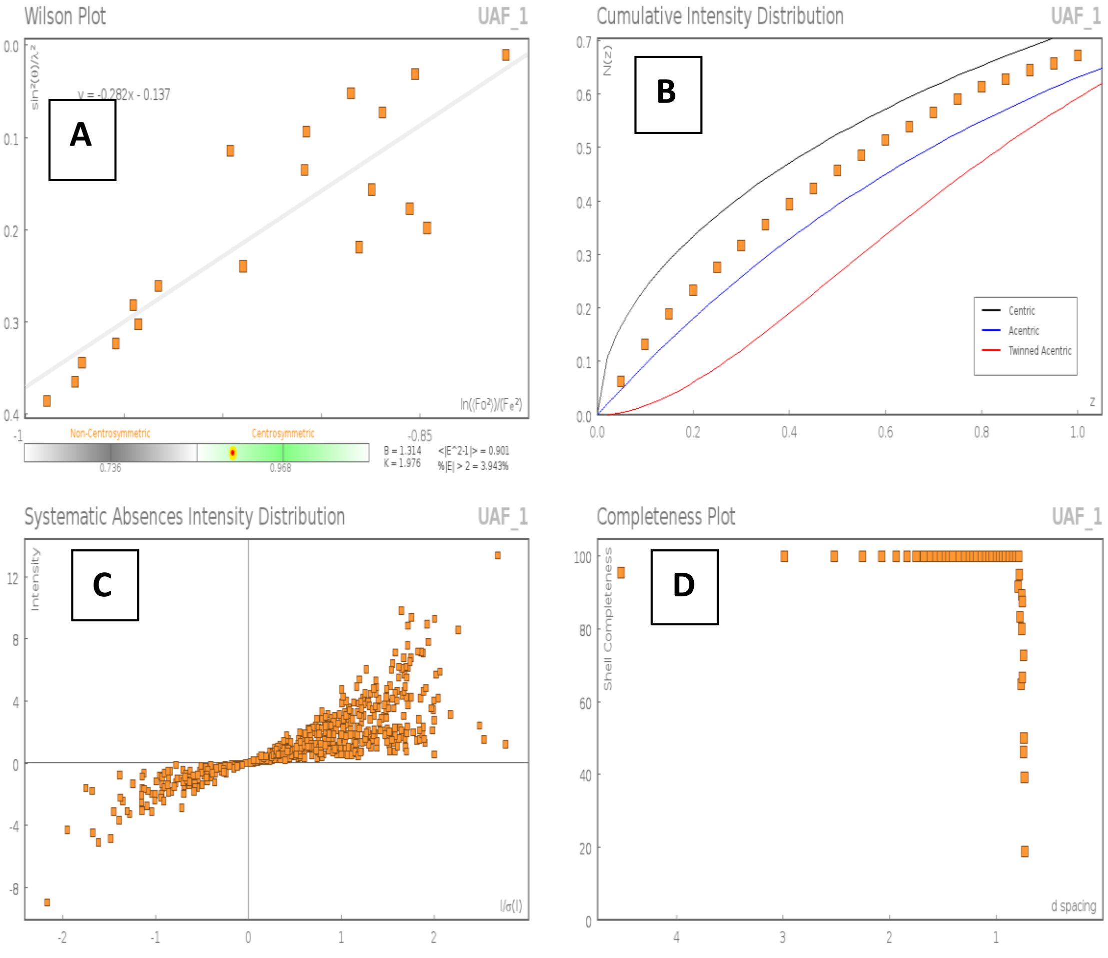

Figure S6. Data plots for diffraction data of compound **1**.

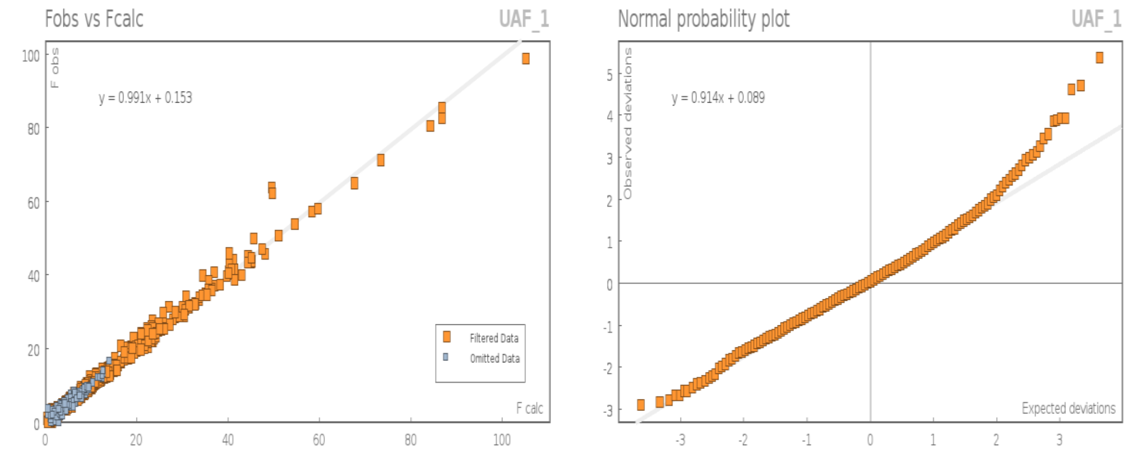

Figure S7. Data plots for refinement data of compound **1**.

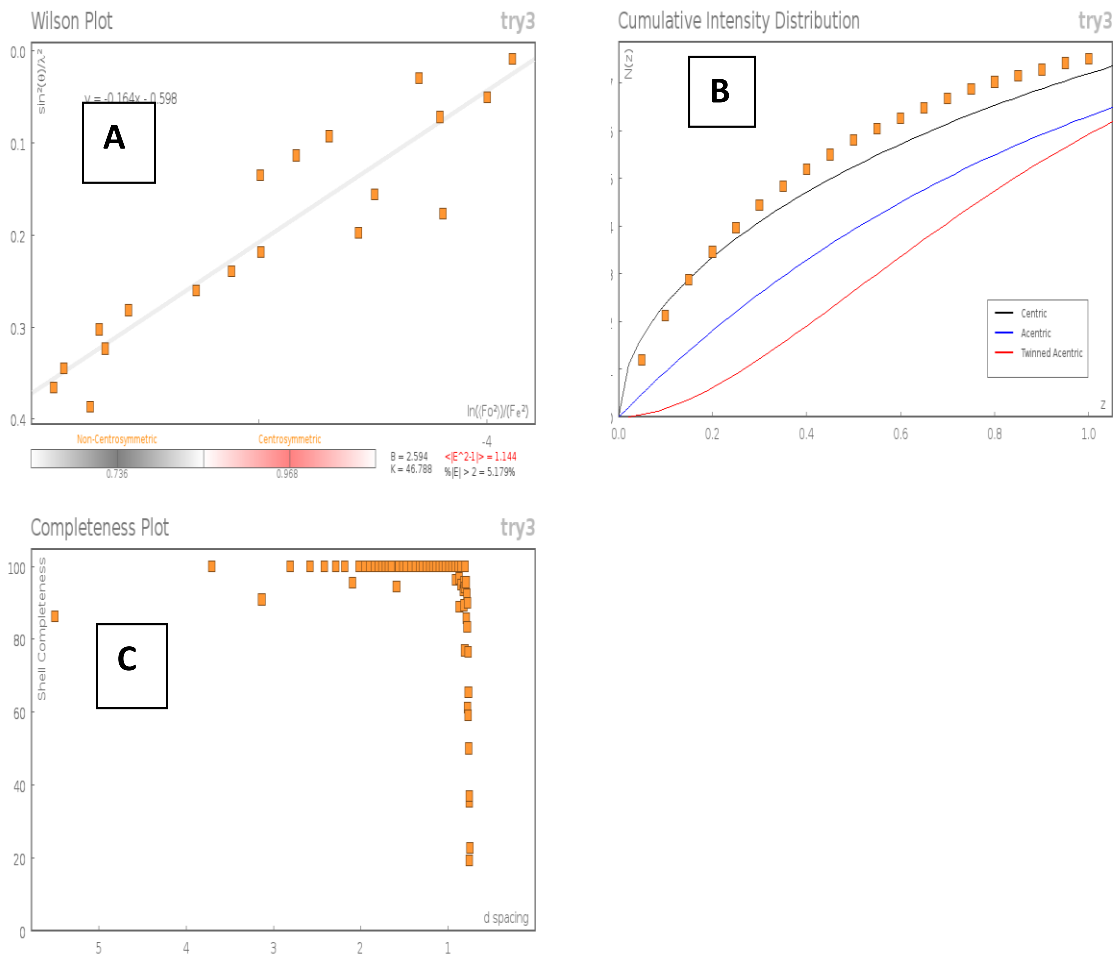

Figure S8. Data plots for diffraction data of compound **2**.

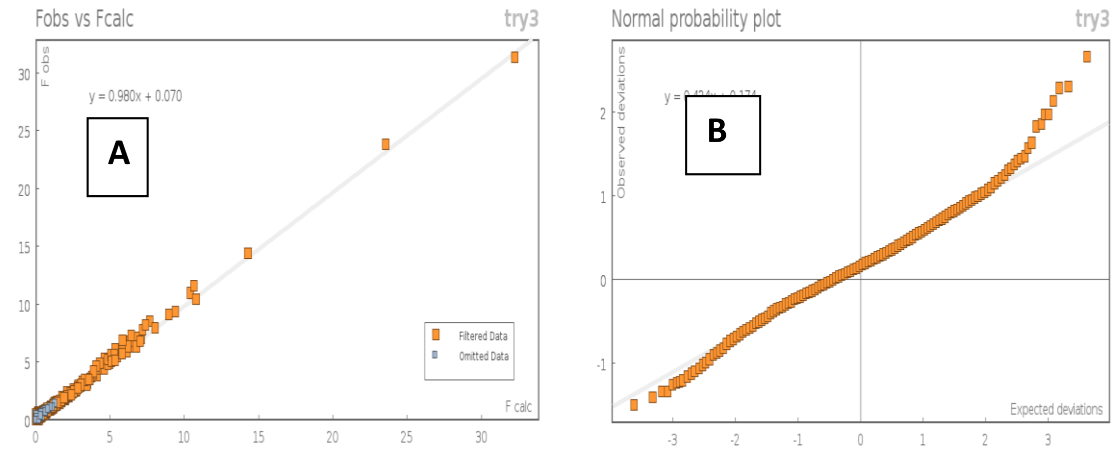

Figure S9. Data plots for refinement data of Compound (**2**).

Table S2. Structure Quality Indicators.

Data	Structure Quality Indicators	Compound (1)	Compound (2)
	d_{min}	0.77	0.77
	1/σ	15.6	11.0
Reflections	R_{int}	4.98 %	2.85 %
	Complete at $2\theta=53^o$	98 %	98 %
	Shift	0.000	0.000
	Max Peak	0.3	0.3
Refinement	Min Peak	-0.3	-0.4
	GooF	0.953	1.170

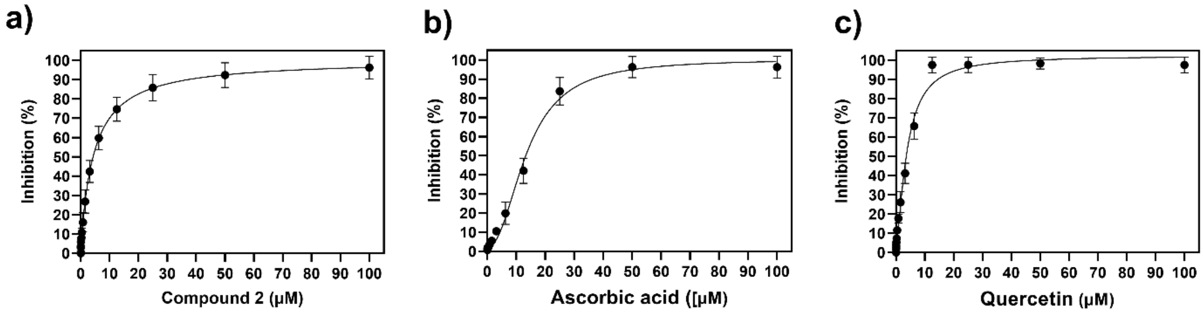

Figure S10. Results of ABTS assay for **a)** Compound **2**, **b)** ascorbic acid**, c)** Quercetin.

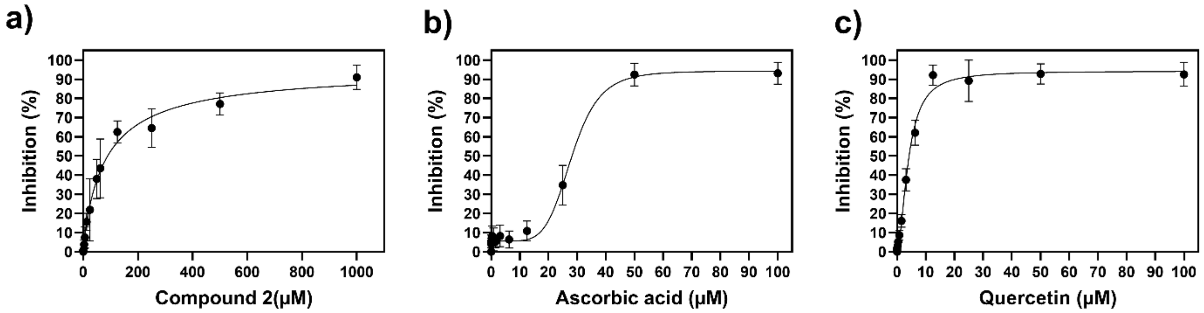

Figure S11. Results of DPPH assay for **a)** Compound **2**, **b)** ascorbic acid**, c)** Quercetin.
